# Welcome to research in health services & regions – methods, results, implementation

**DOI:** 10.1007/s43999-022-00004-4

**Published:** 2022-06-22

**Authors:** Stef Groenewoud, Daniela Koller, Dominik Graf von Stillfried, Thérèse Stukel

**Affiliations:** 1 Radboud universitair medisch centrum, Radboud Institute for Health Sciences, Scientific Center for Quality of Healthcare (IQ healthcare), Nijmegen, The Netherlands; 2 IBE - Institute for Medical Information Processing, Biometry and Epidemiology, Ludwig-Maximilians-Universität München, Marchioninistr. 15, 81377 Munich, Germany; 3 Zentralinstitut für die kassenärztliche Versorgung in der Bundesrepublik Deutschland, Stiftung des bürgerlichen Rechts, Berlin, Germany; 4 ICES, Toronto, Canada; 5 Institute of Health Policy Management and Evaluation, University of Toronto, Toronto, Canada

People should receive the same standard of medical care regardless of where they live or how they access the health care system. Health care systems should guarantee the same quality of care irrespective of geographical location. Despite these aspirations, unwarranted variation in health care has persisted since reported across Vermont by John Wennberg and Alan Gittelsohn more than 50 years ago [1]. This is a problem common to all countries. In the past decade, the Wennberg International Collaborative (https://wennbergcollaborative.org/publications/) has identified unwarranted variations in access to, and performance, of health care in all systems that have been studied.

## Unwarranted variations matter!

Unwarranted variations arise for underuse of effective care, overuse of care where there is excessive supply, and when care fails to meet the preferences of patients [2–4]. While we have ample evidence of unwarranted variations, we need to learn more about effective means of reducing them. This requires a better understanding of the root causes of unwarranted variations and hence how to intervene to reduce them.

## A new journal to discuss variations

Our new international peer-reviewed journal “Research in Health Services & Regions – Methods, Results, Implementation” aims to provide a forum for researchers and health policy professionals focusing on health services research in and for regions. RHSR is published by Springer Nature. We invite scientific papers (including methods and results) and reports of successful health policy interventions. These reports could be on local initiatives that use evidence of geographical variations to implement the redesign of care. We also welcome policy papers on key issues and brief communications such as Research Commentaries and Scientific Letters. RHSR is intended to provide a forum for health services researchers, clinicians and policymakers who are committed to reducing unwarranted variations in health care.

## Themed collections

We aim to publish two or three themed collections every year that are deep dives into current topics. We invite submissions for two themes. The first will be up to 20 papers on “The Future of Health Care Atlases”. We invite those working on Atlas projects to submit a short paper on their approach, experiences, and outlook. The second will focus on “Lessons from the Pandemic – How the pandemic affects our health care system”. This aims to reflect on what we could learn from the COVID-19 pandemic that relates to regional variations in health care.

## Open access

All publications in RHSR will be open access, published under a Creative Commons license, in which authors retain the copyright of their papers. RHSR is sponsored by the Central Research Institute for Ambulatory Health Care in the Federal Republic of Germany which covers Article Processing Charges (APC). No article processing charges or fees will apply. All published papers will be made freely and permanently accessible online, without subscription fees or registration barriers, providing immediate and equitable access to information. Open access content results in increases in citations, downloads, and being mentioned by news media and in policies, ranging from 20% to 300% -- see white paper published by Springer.

## How we work

All manuscripts are peer-reviewed prior to publication in a single-blinded process. Articles are published immediately upon acceptance in a continuous flow.

An international team of renowned specialists is contributing to the journal as members of the editorial board and as peer-reviewers. Get to know them on the journal website under https://www.springer.com/journal/43999/editors.

For advice on articles categories that we accept, please check our journal website: https://www.springer.com/journal/43999/submission-guidelines.

We are looking forward to receiving your manuscripts!
